# The Nurse and Her Newspaper

**Published:** 1918-09-21

**Authors:** 


### THE NURSE AND HER NEWSPAPER.

The sensation of fear is one of the most distressing of human emotions, a fact which every experienced nurse recognises. Fear of pain is worse than the pain itself, and very often the victim is helpless to fight against it, although his intellect convinces him that he is suffering needlessly.

I must confess that I realise with much regret that a very large number of nurses, as well as probationers, suffer from the fear of being found out if they venture to write a letter to their own press. Such, without doubt, finds its origin in ignorance, a fact which I propose this week to demonstrate. Anybody who reads my notes from week to week must realise that a part of my mission is to induce the profession to express itself, to become articulate, and; as though it were a crime to do so, I always find myself up against this nervousness of discovery. Now it is quite apparent that if a nurse speaks above a whisper in some institutions she runs the risk of being marked down as an agitator. A conscientious matron may regard it as a duty to her board, and possibly a pleasure to herself, to reduce such an one to silence. Unfortunately, there are authorities so narrow in their outlook that they never see that other people have a right to be alive, and they absolve themselves from their sins of tyranny by indulging in the belief that they are acting in obedience to the highest dictates of conscience. Their duty, or their supposed duty, to their boards is their apologia for squelching their subordinates. I am not drawing on my imagination. I have it in black and white from many nurses that they are afraid of compromising themselves if they express an opinion on what their conditions of employment ought to cover.

Now I want such as these to realise that a nurse's newspaper is not a trap for the unwary. Every editor occupies a most responsible position—a really great position, and his abuse thereof would be fraught with serious, if not disastrous, consequences. In many respects he may be compared to a banker. Let it be supposed that a shopkeeper is in temporary financial difficulty. He has to pay certain pressing accounts, and is unable to do so at the moment without embarrassment, although he is perfectly solvent. His first and proper impulse is to go to his banker and lay bare his soul. He wants an overdraft. The banker may grant or refuse his request at his discretion. But if the banker dares to disclose that such application is made he may close his doors. Or if the banker's assistant ventures to gossip, even to his intimate friends, the affairs of the bank's customers he does so at the peril of his office. It is a universally recognised principle that confidences between banker and client are sacred. A newspaper is in precisely similar circumstances, and to suggest to an editor that he should disclose the name of a correspondent is to offer him an insult. As well ask a priest to betray the secrets of the confessional. I hold myself a thousand confidences from people whom I have never seen, and apart from any feelings of honour which I may possess I am fully alive to the fact that if I betrayed one of these I should run the risk of never again being trusted by a newspaper editor. I should lose vastly more than my correspondent. I should lose my reputation.

The Hospital is fully alive to its responsibilities. Like the banker, it may grant or refuse a request, but there is no feature of its policy so pronounced as that of preserving inviolate the sanctity of its correspondence. I want this to be thoroughly understood by the humblest and most obscure member of the nursing profession. Nor do I put this forward as a unique virtue. I hope and believe that this applies to the whole British press. Indeed, I do not know of any newspaper to which I should hesitate to write for fear of betrayal by the editor or his staff.

The press is therefore, obviously and naturally, the nurse's vehicle of expression, and she must learn implicitly to trust the editor. She must subscribe her name and address for his personal information, not necessarily for publication. It is this co-operation of mutual confidence, secrecy, and responsibility that makes the newspaper such a power in the land. It has accomplished more for the world than armies and navies. It has brought about bloodless revolutions which have emancipated millions, and is rightly regarded as the chief bulwark of freedom possessed by civilised and free peoples. Speaking for The Hospital, it has helped the College of Nursing to the success which it has already achieved, and its function and duty now is to continue to guide it along the thorny path. But this it can only accomplish in slow and halting fashion if the nurse wraps herself in a veil of silence. Otherwise Matron Browne will accuse us of manufacturing grievances and drawing on our imagination. Already this has happened. The silence of the nurse was interpreted a week or two ago by a correspondent as the outward and visible sign of complete contentment. Fortunately, there was a convincing answer.

A word to the probationer, the sister and the matron of the future. It is her duty to study, and she has many advisers as to text-books and treatises. May I point out to her that above all things she should seek to keep herself in touch with the currents of thought that prevail in her own world? General intelligence is an essential part of the equipment of every student, and this is not to be found in any technical branch of education. It is to be found by observation, by questioning, by analysing, and, more especially, by reading the papers. One paper advocates this policy, another that. Do not adopt either as your own simply because it appears in print. Follow the arguments: think and act according to your convictions, and never, if you can avoid it, allow fear to enter into your soul unless you do something that you know to be wrong. Pursuit after knowledge and freedom is not wrong: it is the privilege and the duty of every British citizen to aim after the highest. Every such effort is a help to others. I am not exaggerating when I state that the most potent engine which the British nurse possesses in her endeavour to progress is her weekly journal. A man may crawl, or he may walk, or pedal a bicycle, or travel in a train—or he may fly. The strength of the engine is the measure of his progress.

IERNE.

